# Coexpression analysis of CD133 and CD44 identifies Proneural and Mesenchymal subtypes of glioblastoma multiforme

**DOI:** 10.18632/oncotarget.3365

**Published:** 2015-01-31

**Authors:** Daniel V. Brown, Paul M. Daniel, Giovanna M. D'Abaco, Andrew Gogos, Wayne Ng, Andrew P. Morokoff, Theo Mantamadiotis

**Affiliations:** ^1^ Department of Pathology, University of Melbourne, Melbourne, Australia; ^2^ Department of Surgery (RMH), University of Melbourne, Parkville, Victoria, Australia; ^3^ Centre for Neural Engineering, University of Melbourne, Parkville, Victoria, Australia

**Keywords:** coexpression, cancer genome atlas, glioblastoma, molecular subtype, cancer stem cells

## Abstract

Accumulating evidence suggests that the stem cell markers CD133 and CD44 indicate molecular subtype in Glioblastoma Multiforme (GBM). Gene coexpression analysis of The Cancer Genome Atlas GBM dataset was undertaken to compare markers of the Glioblastoma Stem-Progenitor Cell (GSPC) phenotype. Pearson correlation identified genes coexpressed with stem cell markers, which were then used to build a gene signature that classifies patients based on a CD133 coexpression module signature (CD133-M) or CD44-M subtype. CD133-M tumors were enriched for the Proneural (PN) GBM subtype compared to Mesenchymal (MES) subtype for CD44-M tumors. Gene set enrichment identified DNA replication/cell cycle genes in the CD133-M and invasion/migration in CD44-M, while functional experiments showed enhanced cellular growth in CD133 expressing cells and enhanced invasion in cells expressing CD44. As with the 4 major molecular subtypes of GBM, there was no long-term survival difference between CD44-M and CD133-M patients, although CD44-M patients responded better to temozolomide while CD133-M patients benefited from radiotherapy. The use of a targeted coexpression approach to predict functional properties of surface marker expressing cells is novel, and in the context of GBM, supports accumulating evidence that CD133 and CD44 protein marker expression correlates with molecular subtype.

## INTRODUCTION

Glioblastoma Multiforme (GBM) is an aggressive, heterogeneous tumor of the central nervous system. The diverse features of the tumor have made management of GBM difficult [1]. GBM has a 5 year survival of less than 5%, rendering it one of the most lethal types of tumors [2]. Molecular profiling of patient specimens has revealed that GBM consists of several distinct subtypes with characteristic mutational, transcriptional and epigenetic profiles. Subtype classification is based on the similarity of the gene expression profiles with the major stages of neural development, Proneural (PN), Neural, Classical and Mesenchymal (MES) [3-5].

GBM is typically treated by a combination of surgical resection, radiotherapy and chemotherapy with temozolomide. Poor patient survival in GBM is due to the recurrence of the tumor despite therapy [6, 7]. It has been suggested that the inevitable recurrence is driven by a subpopulation of GBM cells with stem cell properties, glioma stem-progenitor cells (GSPCs) or glioma initiating cells (GICs) [8].

Understanding the underlying biology of GSPCs relies on the large body of knowledge derived from fundamental stem cell research. Advances in stem cell research has heavily relied on the ability to enrich rare subpopulations of cells for downstream characterization using fluorescence activated cell sorting (FACS) [9, 10]. FACS relies on a robust extracellular protein marker or combination of markers based on prospective characterization of the phenotypic properties of the cells defined by those markers. Therefore the choice of FACS marker is crucial in the experimental design of cancer stem cell studies. GSPCs were initially characterized based on the expression of the stem cell marker CD133 (expressed by the PROM1 gene) [8, 11]. CD133+ cells were demonstrated to exhibit robust cancer stem cell/tumorigenic potential compared to CD133− cells when as few as 100 CD133+ cells are transplanted into immunodeficient mice [12].

Although a number of studies have supported the prognostic value of CD133 in GBM [13-15] there are reports that CD133 expression is not restricted to GSPCs, and that CD133− cells also exhibit stem cell characteristics [16-18]. A variety of other stem cell markers have also been investigated in GBM including the adhesion molecules CD44, Integrin-α6, CD15 (also known as SSEA-1 expressed by the FUT4 gene) and the expression and activity of ALDH1A3 [19-22].

Gene expression profiling experiments show that GSPCs exhibit a similar molecular classification to the parental bulk GBM tumor, with 2 clusters of cells representing the PN and MES subtype [23, 24]. Phenotypically, PN cells exhibit non-adherent sphere forming growth *in vitro* and circumscribed, non-invasive growth *in vivo*. In contrast, MES cells grow semi-adherently *in vitro* and show invasive growth *in vivo* [24, 25]. It has recently been shown that the PN subtype predominantly expresses CD133 or CD15 at the cell surface, whereas the MES subtype expresses CD44 [25, 26].

To determine the precise context of the relationship between the cancer stem cell phenotype, molecular subtype and the expression of extracellular stem cell markers we have used publicly available gene expression data of GBM and GSPC samples to perform coexpression analysis. The utility of coexpression analysis has been previously demonstrated in various cancers, including GBM, through the identification of novel genetic modules, allowing for more precise molecular subclassification of tumor subtypes and the possibility that this information could be used in precision medicine based therapeutic strategies [27-29].

Based on the hypothesis that gene sets/modules coexpressed with specific cell surface markers contribute to the phenotype of the overall tumor, we studied gene signatures derived from the coexpression modules of several stem cell markers. We demonstrate that, in the context of GBM tumor tissue, expression of coexpression modules associated with CD133, CD44 and CD15 mRNA are markers of GBM molecular subtype independent of cancer stem cell molecular signatures.

## RESULTS

### Coexpression analysis of Glioblastoma cancer stem cell markers

To investigate the biological and clinical significance of selected putative cancer stem cell markers (Table 1) in GBM, a coexpression analysis was undertaken using The Cancer Genome Atlas (TCGA) Agilent microarray dataset. The Agilent dataset (483 patients) demonstrated more normally distributed gene expression profiles compared to the Affymetrix U133a dataset (539 patients) (Figure S1A and B). The top 5% of significant positively correlated genes (332-674 genes in length) with each cell surface marker mRNA was used to build a coexpression module (Table S1).

**Table 1:** GSPC markers selected for analysis

Marker	Function	Reference
CD133 (PROM1)	Unknown biological function	[8]
CD44	Hyaluronan binding	[19]
CD15 (FUT4)	Transfer fucose to polysaccharides	[20]
Integrin-α6	Subunit for laminin receptor	[22]
L1CAM	Cell-cell adhesion in neural lineage	[21]
ALDH1A3	Detoxification of aldehydes generated by metabolism	[37]

Positively correlated genes were selected for the signatures as they are expressed in the population with the stem cell marker and therefore are able to be detected, unlike negatively correlated genes.

The CD133 module signature (CD133-M) was negatively correlated with CD44, whereas the CD44 and CD15 module signatures (CD44-M and CD15-M) were highly correlated with each other (Figure S1C and D). It is interesting to note that there are no genes that are positively correlated with both CD133-M and CD44-M.

The greater overlap of CD44-M and CD15-M with the MES subtype was likely due to the greater magnitude of the Pearson correlation coefficients for genes coexpressed with CD44 mRNA compared to CD133 mRNA (Figure S1E), due to higher absolute expression of CD44 mRNA in the GBM tumors (Figure S2A). As recent reports suggest a subset of cancer stem cell markers enrich for characteristic GBM molecular subtypes [25, 26], coexpression modules were compared to the assigned molecular subtype for each patient. The TCGA RNAseq GBM dataset was utilized as an independent technical platform from the Agilent array dataset, to investigate association with molecular subtype.

**Figure 1:**
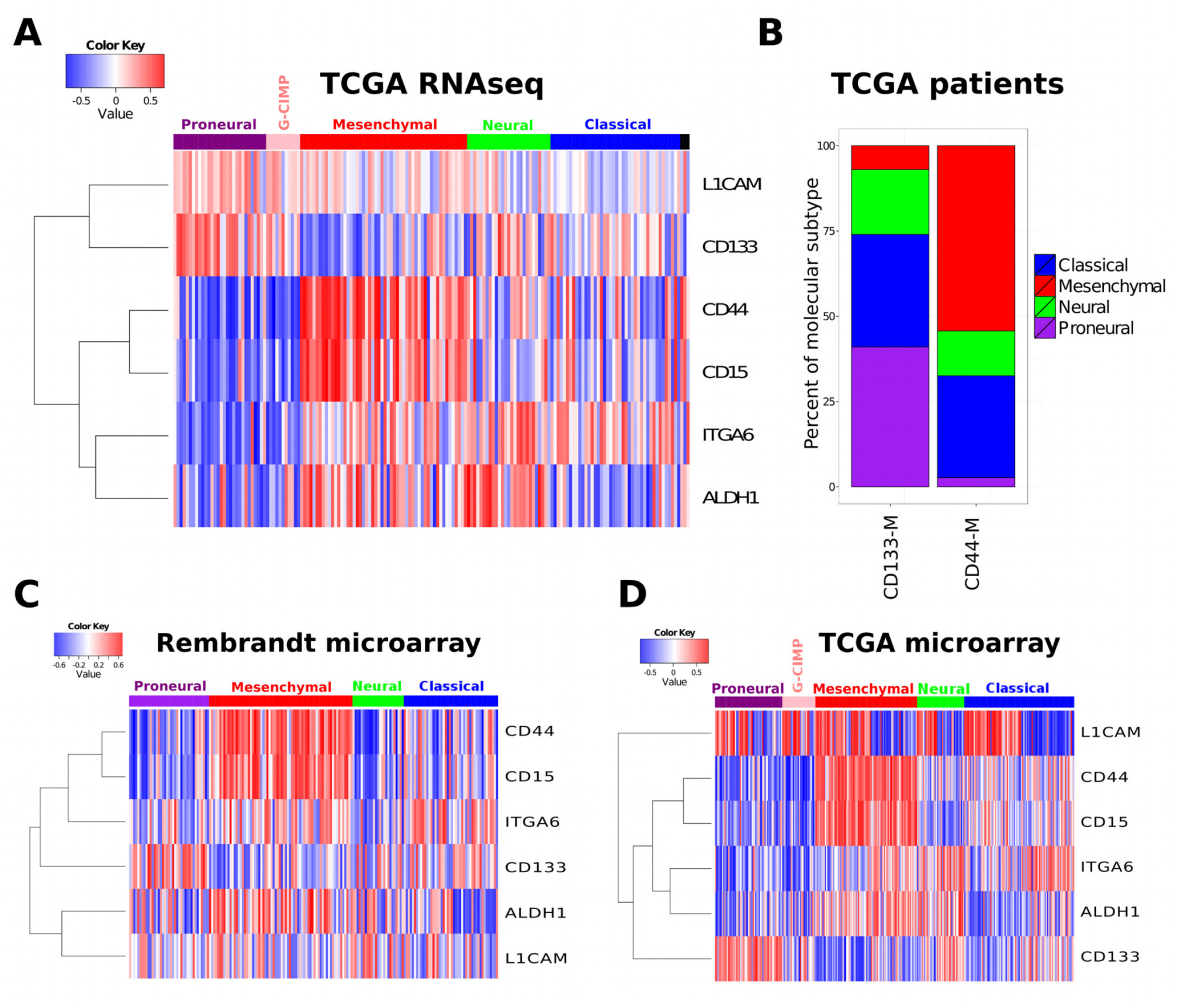
Cancer stem cell marker coexpression modules are associated with molecular subtype (A) The coexpression module signatures of a range of stem cell markers was used to assign a patient specific similarity score. This score was then related to the assigned molecular subtype (top horizontal bar). (B) Enrichment of PN subtype in CD133-M and enrichment of CD44-M in MES. All patients in the 2013 data freeze were assigned to either a CD133 or CD44 coexpression module subtype (CD133-M or CD44-M) and the percentage of each molecular subtype within each coexpression subtype calculated. Coexpression module signature analysis of the Rembrandt (C) and (D) and the TCGA Agilent microarray dataset.

The coexpression modules derived from CD133, CD44 and CD15 mRNA expression showed a striking pattern of overlap with the two most distinct molecular subtypes, PN and MES (Figure 1A). CD133-M was highly enriched in the PN molecular subtype (p-value 4.2 e-08, Wilcoxon rank sum test). The number of genes shared between CD133-M and PN signatures was also significant at 31 genes (p-value 8.6e-16, hypergeometric test) (Table S2). Conversely CD44-M was enriched in the MES subtype (p-value 9.7e-14) and the number of genes that overlapped was greater at 106 genes (p-value 1.3e-106), (Figure 1A). The number of genes shared between CD15 and MES signatures was 97, (p-value 5.93e-91), slightly smaller than for CD44-MES (Table S2). Given the redundancy (overlap) for CD44-M and CD15-M in marking the MES molecular subtype in our analyses, we focused primarily on a CD133/CD44 classification based on the coexpression modules derived from these markers.

Although the CD133-M/CD44-M classification separates PN from MES molecular subtypes the distribution of CD133-M/CD44-M was not significant in Classical tumors (p-value 0.53, binomial test) or Neural tumors (p-value 0.85, Figure 1B). The enrichment of CD133-M, CD44-M and CD15-M was reproduced in the independent Rembrandt dataset (Figure 1C) and the original Agilent TCGA dataset used in the coexpression analysis (Figure 1D).

Other markers tested showed a more scattered overlap with molecular subtype. Of note, the ALDH1A3 module showed an association with the MES subtype as previously reported (p-value 1.5e-4) [25].

The Integrin-α6 module was lower in the PN subtype compared to other subtypes, while the L1CAM marker was inconsistent in the Rembrandt dataset compared to the TCGA (Figure 1C). No marker tested could discriminate the G-CIMP subgroup of PN GBMs from the non G-CIMP PN subtype.

Coexpression analysis was also performed with the addition of intra-cellular markers related to neural differentiation (Figure S2C). The neural marker TUBB3 (also known as β3-tubulin) and oligodendrocyte marker OLIG2 were associated with the PN subtype, as previously reported [25, 26]. Conversely the astrocyte marker GFAP and mesenchymal marker YKL40 were associated with the MES subtype.

There were 3 broad clusters of coexpression modules that were associated with the GBM samples. One cluster was indicative of the MES subtype, another of PN while a third contained markers of neural immaturity including nestin, Pax6 and ID1. Our data suggests that coexpression module signatures identify GBM molecular subtype better than either raw mRNA expression (Figure S2A) or z score normalized mRNA expression in the TCGA (Figure S2B).

### The coexpression module subtypes of GBM are reflected in glioma stem-progenitor cell cultures

**Figure 2:**
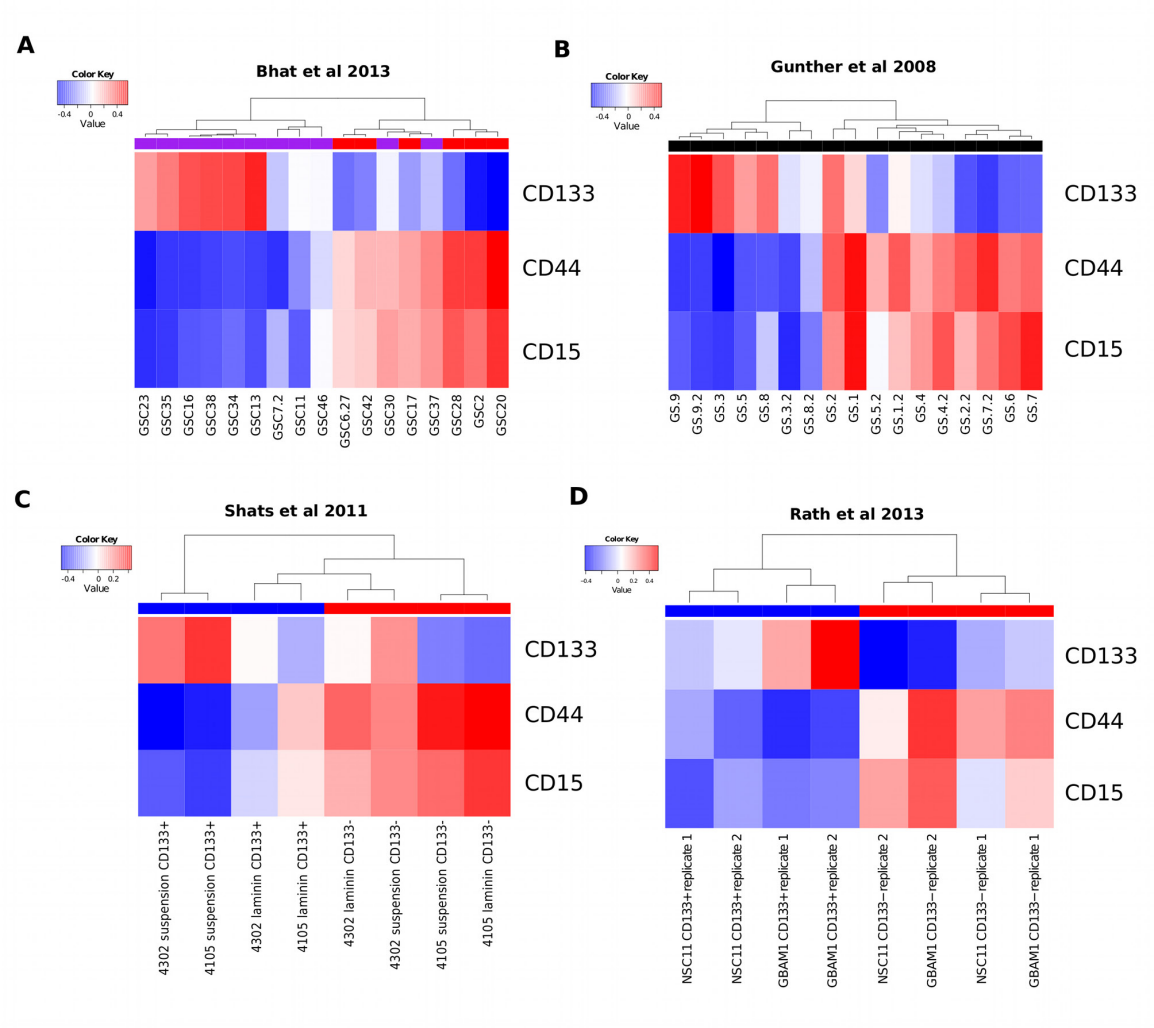
The coexpression modules identified in GBM tumors are reflected in GSPCs (A) Coexpression module analysis of Bhat el al 2013 gene expression data. Hierarchical clustering of GSPC cultures by CD133-M and CD44-M partitions samples into 2 groups, largely overlapping with the assignment of PN (purple horizontal bar) and MES (red horizontal bar). (B) Coexpression module analysis of Gunther et al 2008 reveals 2 groups by clustering that largely overlap with the suspension or semi-adherent growth pattern described in the study. (C) Coexpression module analysis of Shats et al shows that CD133+ (blue) sorted cells are assigned to the CD133 coexpression signature and CD133− cells (red) are assigned to the CD44 coexpression signature. (D) Coexpression module analysis of Rath et al shows both normal neural and GBM cells that are CD133+ (blue) are enriched for CD133-M and CD133− cells are enriched for CD44-M (red).

We next investigated if glioma stem-progenitor cells (GSPCs) grown *in vitro*, reflected the coexpression modules identified using whole tumor derived gene expression measurements. Analysis of publicly available datasets confirms the mutual exclusivity of CD133-M and CD44-M (Figure 2A and B). For the gene expression study conducted by Bhat et al., (2013) the PN subtype assigned by the authors overlapped with CD133-M and the assigned MES signature overlapped with CD44-M (Figure 2A) [26]. Likewise for the Gunther et al. (2008) dataset the coexpression modules overlapped with the growth pattern of the GSPCs described in the study, suspension for PN cells and semi-adherent for MES cells with the exception of 1 sample [30].

To investigate if CD133-M is enriched in CD133 protein expressing cells we reanalyzed the Shats et al. (2011) dataset which was based on gene expression profiling of CD133 sorted cells [31] (Figure 2C). As expected, CD133+ cells grown in suspension culture conditions were strongly positive for CD133-M. However, CD133+ cells grown on the adherent substrate laminin were more strongly associated with CD44-M, suggesting a shift towards a more MES-like subtype in adherent culture conditions. This is consistent with the role of CD44 in the binding of extracellular matrix molecules such as laminin [32]. Overall, CD133− cells were associated with CD44-M, consistent with the pattern observed in a second dataset that included both normal neural stem cells and GSPCs (Figure 2D) [33].

### Coexpression analysis identifies pathways involved in tumorigenesis

**Figure 3:**
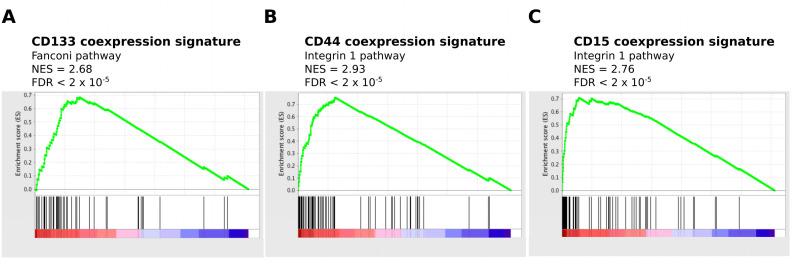
Enrichment of proliferative pathways for CD133-M and enrichment of invasive pathways for CD44-M and CD15-M The Pearson correlation score of all genes with CD133, CD44 and CD15 mRNA was used as input to pre-ranked GSEA. (A) Enrichment of proliferative pathways in CD133 correlated genes. (B) and (C) Enrichment of invasive pathways for CD44 and CD15 correlated genes.

We next investigated pathways associated with the identified coexpressed genes by gene set enrichment analysis (GSEA). The Pearson correlation coefficient of all genes on the Agilent platform with CD133, CD44 and CD15 mRNA were used as input to GSEA [34]. Pathways highly correlated with CD133 mRNA expression included those involved in DNA repair, cell cycle and DNA replication (Figure 3A and Table 2). This suggests that tumors with high CD133 expression are more proliferative than tumors with low CD133 expression. This is more consistent with a progenitor or transit-amplifying cell, and not a quiescent stem cell. In contrast, GSEA of the intracellular stem cell marker Pax6 revealed an association with the Notch pathway, while expression of the neural stem/progenitor marker nestin, was associated with an active Hedgehog pathway (Figure S3). Notch signaling has a role in the maintenance of stem cell identity [35] while the hedgehog pathway is involved in the regulation and identity of adult neural stem cells [36].

**Table 2:** Summary of functional categories enriched in genes coexpressed with GSPC markers

CD133	CD44	CD15
Fanconi pathway	Integrin1 pathway	Integrin1 pathway
G2/M checkpoints	Cytokine receptor pathway	Extracellular matrix organization
Cell cycle	NOD-like receptor pathway	Integrin3 pathway
Meiosis	Complement and coagulation cascades	Focal adhesion
Chromosome maintenance	Interferon gamma signaling	ECM receptor interaction
DNA replication	Integrin3 pathway	Leukocyte transendothelial migration
Double strand break repair	ECM receptor interaction	TRAIL pathway
Packaging of telomere ends	NFκB pathway	P53 downstream pathway

The validity of the analytical approach is strengthened as the top pathways enriched for each marker often reflected the known biological role of the protein in question. ALDH1A3 is involved in the oxidation of intracellular aldehydes including retinol, producing retinoic acid [37]. The pathways associated with CD44 and CD15 included those involved in invasion and migration (Figure 3B and C). These pathways are consistent with the previously described role of CD44 as a MES marker in multiple cancer types [38]. In other cases there was no clear relationship between the pathways enriched by coexpression analysis and the stem cell marker, as for L1CAM and Sox2 (Figure S3).

### Functional validation of predicted signatures enriched in CD44 and CD133 expressing GSPCs

**Figure 4:**
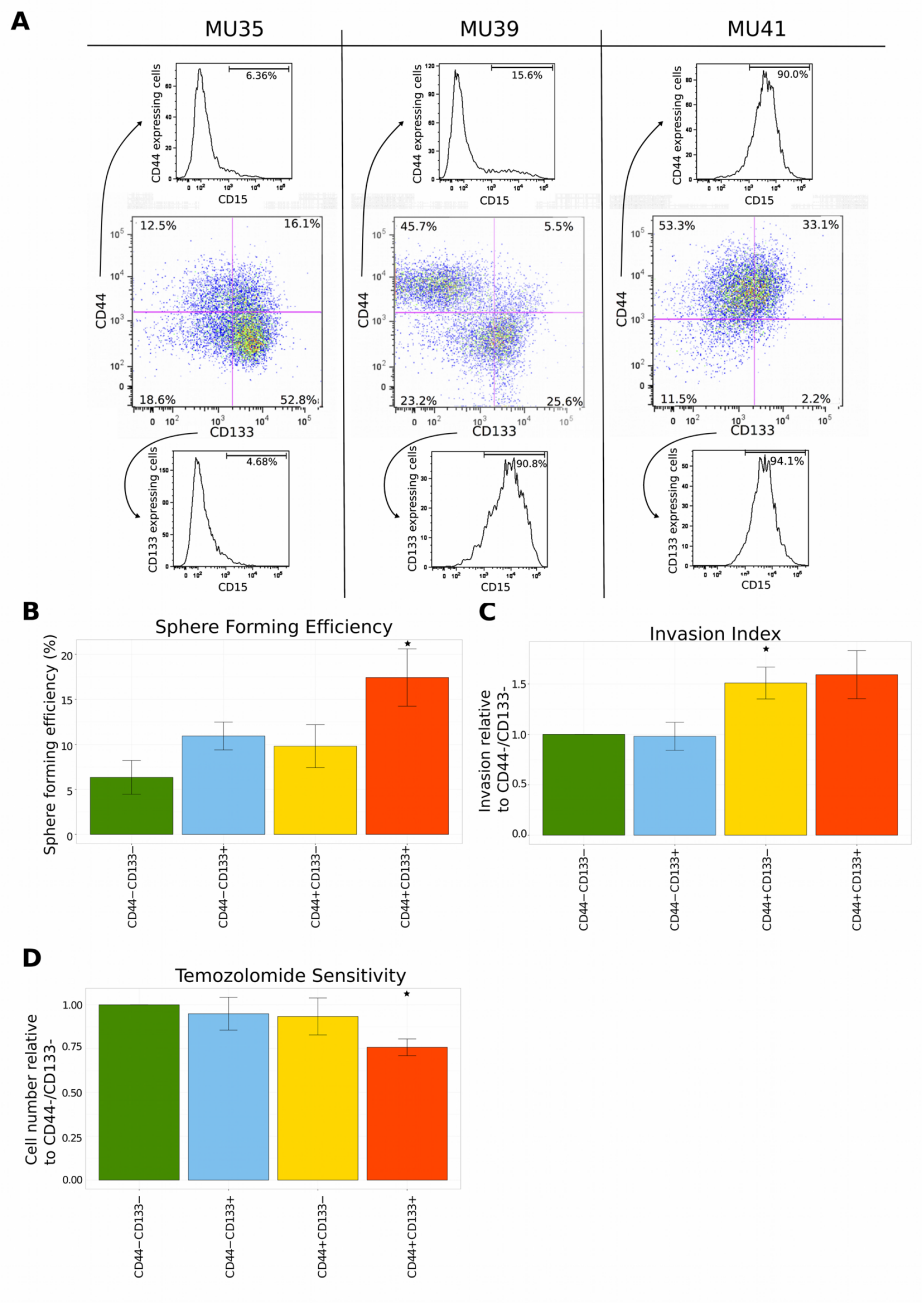
Functional validation of the coexpression module and pathway analysis (A) FACS analysis of the expression of surface protein markers using a panel of GSPCs. Cells were labeled with CD15, CD44 and CD133 antibodies. Expression of CD44 and CD133 is represented in the center panel. The expression of CD15 is shown separately for CD44+ cells (top) and CD133+ cells (bottom). (B) Repopulation ability is highest for CD44/CD133 double positive GSPCs. Limiting dilutions of cells ranging from 50 to 1 cell per well were sorted into plates and scored for sphere formation after 7 days. Sphere forming efficiency was estimated by ELDA. Error bars represent sem of three independent GSPC lines. (C) CD44 expressing GSPCs are a more invasive subpopulation. The surface area of the sorted GSPCs was quantified 7 days after the administration of extracellular matrix. Mean invasion is represented relative to the CD44−/CD133− subpopulation. Error bars represent sem of five independent GSPC lines. (D) Temozolomide sensitivity of sorted GSPCs was measured 7 days after sorting. 50μM of temozolomide was applied daily. Mean temozolomide sensitivity is presented relative to the CD44−/CD133− subpopulation. Error bars represent sem of five independent GSPC lines.

To verify the coexpression analysis experimentally, functional assays were performed to test the hypotheses generated by gene set enrichment. The cell surface protein expression of CD15, CD44 and CD133 was examined in a panel of GSPC lines (‘MUxx’), derived from patient tumors using flow cytometry (FACS). Similar to the coexpression module signatures there was a bias towards the expression of either CD133 (MU35), or CD44 (MU39 and MU41) (Figure 4A). However the relationship between expression of CD15 with CD44 or CD133 was not clear. For GPSC line MU39 CD15 was coexpressed with CD133. However MU35 did not express CD15 and MU41 expressed CD15 throughout the population (Figure 4A). It is of note that MU39 is unique in that there are 2 distinct subpopulations that express CD44 and CD133 respectively.

FACS sorted cells were examined for a range of phenotypes predicted by GSEA. Interestingly CD44+/CD133+ cells had a significantly increased growth and self-renewal capacity demonstrated by the ability to form gliomaspheres at low dilution, a property of more stem-like cells (p-value 0.030, Tukey post-hoc test following ANOVA), (Figure 4B). Cells expressing CD44 had a greater invasive ability with CD44+/CD133− GSPCs exhibiting a significantly increased invasion index compared to CD44−/CD133− cells (p-value 0.007, Tukey post-hoc test), (Figure 4C). CD44+/CD133+ GSPCs were more sensitive to temozolomide, in contrast to cells that expressed CD44 or CD133 alone (p-value 0.041, Tukey post-hoc test), (Figure 4D).

As proliferation and invasion are the phenotypes predicted by GSEA for CD44-M and CD133-M, the functional examination of CD44 and CD133 expressing cells *in vitro*, (Figure 4) validates the targeted coexpression approach to computationally assess cell surface markers used for flow cytometry.

### Examining the relationship between coexpression module signature and genomic alterations

To further investigate the molecular differences between CD133-M and CD44-M classified patients, exome sequencing data was used to compare the overall number of somatic mutations between the 2 groups. In contrast to previously published findings [39], there was no difference in the total number of mutations for the CD133-M subtype compared to the CD44-M subtype (Figure S5A).

Pairwise testing for mutated genes enriched in each subgroup revealed that the CD133-M subtype was strongly enriched for IDH1 mutation with an odds ratio (OR) of 10.9, (p-value 5.91e-05, Fisher's exact test), (Figure S5B). TP53 mutations were also enriched (OR 2.60, p-value 0.0127). The NF1 gene which is characteristic of the MES subtype, was slightly above the threshold for statistical significance in the CD44-M subtype (OR 2.94, p-value 0.059). There was no other significant difference in genes commonly mutated in GBM, including PTEN, PIK3CA, PDGFRA and EGFR.

As epigenome status is intimately linked to gene expression, the relationship between the coexpression module subtypes and promoter methylation was examined. The CD44-M subtype was significantly associated with the M1 subtype (p-value 0.021, binomial test with FDR [False Discovery Rate] correction). This is consistent with the M1/MES molecular subtype enrichment previously described [5]. The CD133-M subtype was significantly enriched in the G-CIMP and M6 subtypes, methylation subgroups that are hyper and hypo- methylated respectively (p-value 10e-4 for both G-CIMP and M6 ). There was also a subtle but significant enrichment of CD133-M subtype in M3 (p-value 0.041) and CD44-M subtype in M2 (p-value 0.049). However, there was no difference in the methylation status of the MGMT promoter between the two groups (Figure S5B).

It has previously been reported that CD133 mRNA expression is negatively associated with IDH1 mutation and the G-CIMP group of Proneural GBMs [40]. Our analyses confirm this observation (p- value 0.006, t-test) (Figure S5C). However it is interesting that there was no significant difference in CD133-M between G-CIMP patients and non G-CIMP patients. This indicates that G-CIMP patient tumors share a gene expression profile similar to CD133-expressing tumors overall, despite a lack of CD133 mRNA expression.

Overall, the relationship between somatic mutations, methylation and coexpression subtype remains consistent with the notion that CD133-M is a marker of the PN molecular subtype and CD44-M identifies the MES subtype.

### CD44 and CD133 coexpression signatures predict temozolomide and radiation response

**Figure 5:**
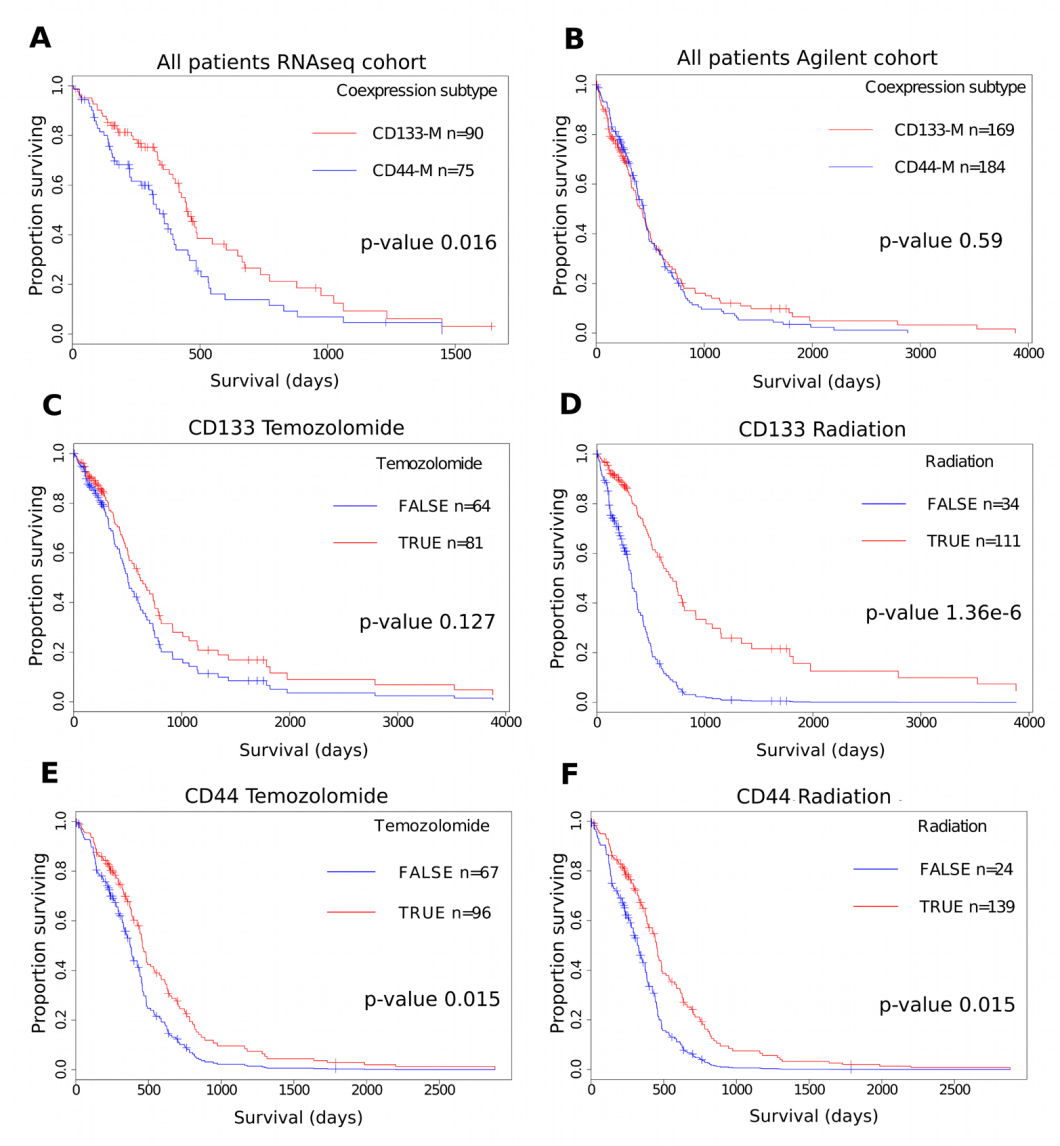
Differential response to therapy for CD44-M and CD133-M classified patients in the TCGA Agilent cohort (A) Survival advantage for CD133-M classified patients in the TCGA RNAseq cohort. (B) Survival analysis of the TCGA Agilent microarray GBM dataset. CD133-M patients receive greater benefit from radiotherapy. CD133-M classified patients in the Agilent cohort were stratified on the basis of receiving temozolomide (C) or radiotherapy (D). All other covariates are set to the mean of the dataset. CD44-M patients receive greater benefit from temozolomide. CD44-M classified patients were stratified on the basis of receiving temozolomide (E) or radiotherapy (F).

Due to the observed overlap between CD133-M and the PN subtype and CD44-M and CD15-M with the MES subtype, we investigated the utility of a dichotomous CD133-M/CD44-M classification to predict survival. As the number of genes coexpressed with both CD44 and CD15 was 30.4% (p-value 2.95e-144, hypergeometric test), we decided to focus on CD133 and CD44 coexpression module signatures in the survival analyses. Patients were classified as belonging to either CD133-M or CD44-M subtypes based on their gene set signature scores as computed by GSVA [41] (see methods). For the set of GBM patients assayed on the RNAseq platform, (TCGA) CD133-M subtype patients had a significant survival advantage compared to CD44-M subtype patients (p-value 0.016, log-rank test) (Figure 5A).

However there was no statistically significant difference in survival between CD133-M and CD44-M patients in either the Agilent cohort or the Rembrandt cohort of patients (Figure 5B) and (Figure S6). We could not identify any factors to explain the difference in survival between RNAseq and Agilent platforms aside from the timeframe within which the samples were collected (1989 - 2011 for Agilent microarray) and (1998 - 2011 for RNAseq). That there was no overall survival difference between CD44-M and CD133-M patients in Agilent and Rembrandt datasets is not unexpected as G-CIMP samples aside, there is no long-term difference in survival for the 4 major molecular subtypes of GBM [5].

There were however, differences in response to therapy for CD133-M and CD44-M classified patients in the Agilent cohort (Figure 5C-F). To investigate these factors further, a Cox proportional hazards model was constructed separately for CD133-M and CD44-M patients, incorporating age, G-CIMP status and exposure to radiotherapy and temozolomide (Table 3). CD133-M patients had significantly improved survival from radiotherapy treatment (95% CI hazard ratio 0.173 - 0.477) but no significant benefit from temozolomide (HR 0.474 - 1.097). In contrast, CD44-M patients received a greater survival benefit from temozolomide treatment (HR 0.416 to 0.908) and a greatly reduced benefit from radiotherapy (HR 0.298 to 0.880), compared to CD133-M patients.

**Table 3:** Hazard ratios for CD133-M and CD44-M GBM subtypes by treatment

Subtype	Treatment	Hazard ratio (95% CI)	p-value
CD133-M	Temozolomide	0.722 (0.474 to 1.097)	0.127
CD133-M	Radiation	0.287 (0.173 to 0.477)	1.36e-6
CD44-M	Temozolomide	0.615 (0.416 to 0.908)	0.0145
CD44-M	Radiation	0.512 (0.298 to 0.880)	0.154

## DISCUSSION

This study is the first to demonstrate an association between cell surface markers and molecular subtype using gene expression profiles derived from large patient cohorts. Both TCGA and Rembrandt patient cohorts, as well as RNAseq and microarray technical platforms identified this association. Coexpression modules associated with putative cancer stem cell markers were identified and related to recognized molecular subtypes of GBM and clinical outcome. There was a significant overlap of CD133-M with the PN subtype and conversely CD44-M with the MES subtype. Association of these cell surface markers with these molecular subtypes has been previously demonstrated in both cell cultures and patient specimens using FACS and IHC [22, 23].

GSEA predicted DNA replication and cellular growth pathways as enriched in CD133-M subtype tumors and invasion and migration pathways as enriched in CD44-M tumors. This was validated functionally using a panel of GSPCs with CD44+ cells being more invasive and CD44+/CD133+ cells being more sensitive to temozolomide.

Coexpression module analysis of Pax6 and nestin showed enrichment of the Notch and Hedgehog pathways respectively, supporting the use of these markers for identifying stem/progenitor cells and validating the targeted coexpression approach for predicting phenotypes associated with markers of interest. However, a limitation of using FACS for functional characterization of marker expressing cells is the requirement for localization of the epitope at the cell surface; both Pax6 and nestin are nuclear proteins and cannot be used for live cell sorting by antibody based methods.

A limitation of our study is the use of mRNA as a surrogate for the expression of cancer stem cell markers. It has recently been demonstrated that there is only a modest correlation between mRNA abundance and protein expression for TCGA tumor specimens [42]. CD133 also undergoes extensive mRNA splicing, generating at least 28 isoforms [43]. The CD133 antibodies used to identify cancer stem cell subpopulations are raised against the glycosylated epitope, which is susceptible to changes in cell cycle phase and oxygen tension [44]. Likewise for CD44, multiple isoforms and glycoslyation states exist [45, 46]. The CD44v isoform is particularly associated with cancer and metastasis [45]. It is primarily expressed on epithelial cells, during embryonic and hemopoietic development [47]. CD44v is also upregulated in GBM [48]. The microarray measurements used to generate the coexpression signatures do not capture isoform information. Isoform level classification of GBM into molecular subtypes has been demonstrated to improve the power to resolve differences in survival [49].

Although the original description of PN and MES molecular subtypes indicated a survival advantage for PN tumors, the discovery of the G-CIMP subgroup of GBM revealed that G-CIMP negative PN tumors had the worst survival of all GBM subgroups [4, 5, 50]. The observation that CD133-M patients receive more benefit from radiotherapy and CD44-M patients receive greater benefit from temozolomide, independent of G-CIMP status is interesting. That CD133 coexpressed genes were involved in cell cycle and DNA replication suggests CD133-M patients would be more susceptible to therapies targeted to highly proliferative cells. The observation that CD133-M patients receive less benefit from temozolomide suggests more complicated mechanisms are involved. Tumors associated with high CD44 expression are known to be more invasive, therefore it is not surprising that it is more difficult to target these tumor cells by radiotherapy. Coexpression analysis indicates that CD15-M is a MES marker. In contrast, functional experiments indicate that CD15 protein expressing cells behave like PN cells [26].

Our study is the first to report triple FACS labeling of GSPCs and did not clarify the relationship of CD15 surface protein expression with CD44 and CD133. Given the small size of our dataset it is difficult to integrate CD15 surface protein expression in the PN-MES spectrum, in the 3 GPSC lines analyzed there was a strong genotypic effect with respect to CD15 expression (Figure 4A)

Therefore the utility of CD15 as a PN or MES marker remains open for further investigation.

It is interesting to note that in our computational analysis of CD133 and CD44 makers there was significant mutual exclusivity in coexpression module signatures. The gene expression measurements in the TCGA represent the average of the tumor population, therefore the mutual exclusivity of the CD133-M/CD44-M signature we have described may be an over simplification. We have not taken into account in our analysis the presence of alternate minor molecular subtypes in a GBM tumor [51, 52].

This may be the reason why there was greater cellular heterogeneity with respect to surface protein expression of CD133, CD44 and CD15 (Figure 4A).

Given that the cancer stem cell theory suggests that stem cells are a rare subpopulation, it is possible that measurements we have used to generate the coexpression signatures do not capture the transcriptome of the GSPCs *in vivo*.

It has recently been established that in GBM, the PN subtype is the original or ground-state subtype and the other molecular subtypes, including MES are descended from a genetic lineage that arises by clonal evolution. Upon clinical presentation there is generally a dominant subclone which contributes the majority of the gene expression signal [53]. This may be the reason for the mutually exclusive nature of the coexpression subtypes.

The analysis and data generated in this study suggest that CD133 is a marker that enriches for the subpopulation of PN cells that reside in GBMs. PN tumors have been shown to grow more efficiently *in vitro* and have a greater sphere forming ability [26] and (Figure 4B). In contrast to these more aggressive *in vitro* characteristics, PN cells form more circumscribed tumors *in vivo*. MES cells grow more slowly *in vitro* but are more invasive and vascular *in vivo* (Figure 4C) and [24]. Therefore the original observations of CD133 expressing cells suggest stem-like properties but may in fact represent the selective advantage these cells have for *in vitro* conditions. MES cells are more aggressive *in vivo*, therefore therapies need to target both these infiltrating cells and the progenitor-like PN cells.

The results presented here can assist in the design of phenotypic marker panels for deciphering the cellular heterogeneity inherent in GBM. This study also provides evidence of the utility of molecular subtyping of GBMs along an axis of PN and MES using the expression of CD133 and CD44 protein, thereby providing a technically simple and economical approach for subtyping patient GBM specimens.

## METHODS

### Source data

The analyses performed in this study are in part based upon data generated by the TCGA Research Network [4, 5]. Level 3 RNAseq and Agilent microarray data was obtained from the UCSC cancer genome browser on May 14 2014 [54]. The Cancer browser in turn obtains processed data from the Broad Institute Firehose pipeline at http://gdac.broadinstitute.org/. Patients that were profiled on both RNAseq and Agilent platforms were removed from the Agilent and retained in the RNAseq dataset. An independent GBM data set was accessed from the Rembrandt database on May 21 2014 [55]. Molecular subtype per patient was assigned by GSVA using established molecular signatures [4]. The source data from Bhat et al 2013 was downloaded from the Gene Expression Omnibus (GEO) accession number GSE49009, Gunther et al. from GSE8049, Shats et al. from GSE24716 and Rath et al. from GSE63037 [26, 30, 31, 33]. Raw affymetrix CEL files were normalized by the RMA algorithm [56]. Probes values were summarized to a single gene measurement by the average reps function in limma (Linear Models for Microarray Data) [57]. The methylation subgroup was retrieved from the TCGA GBM Oct 2012 data freeze, https://tcga-data.nci.nih.gov/docs/publications/gbm_2013/TCGA_GBM_dnameth_scores_20120112_ver3.txt. G-CIMP, IDH1 and MGMT status was obtained from table S7 of Brennan et al. [5]. For all TCGA data the mBatch tool, http://bioinformatics.mdanderson.org/tcgambatch/ was used to inspect for any batch effects. No significant batch effects were observed for the datasets used in this study.

### FACS marker coexpression module signature discovery

The ‘corAndPvalue’ function from the weighted gene coexpression network analysis package was used to compute the Pearson correlation and p-value under the null hypothesis of the correlation being 0 [58]. Pearson and Spearman correlation derived highly similar results; therefore Pearson correlation was used to compute pair-wise gene correlation (Figure S1G and S1H). A cutoff of 2 standard deviations above the mean correlation value and FDR corrected p-value less than 0.05 was used to select the genes for each coexpression module.

### Coexpression module signature score computation per patient

To assign a sample specific score based on enrichment of the derived coexpression modules, the GSVA algorithm was used [41]. GSVA is a non-parametric gene set enrichment method that computes a sample level statistic based on the Kolmogorov-Smirnov random walk statistic. The enrichment score was calculated using the magnitude of the difference between the largest positive and negative random walk deviations. This approach favors genes in pathways that are concordantly activated in one direction only, ie overexpressed relative to the remainder of genes not in the set, as is the case for the standard GSEA algorithm. The coexpression module subtype was designated on a individual patient basis by comparing the enrichment scores for CD133-M and CD44-M and assigning a subtype based on the greater value (Figure S1F).

### Gene set enrichment of coexpressed genes

Gene set enrichment analysis was carried out with the java based GUI version 2.2.0.13 [34]. Genes coexpressed with extracellular markers were ranked by the Pearson correlation value and analyzed in pre-ranked mode. Gene set permutation was used to assess statistical significance. The MolSigDB pathway databases interrogated were C2: Canonical Pathways (KEGG, Reactome, Biocarta and PID) for a total of 4722 gene sets.

### Cell culture and FACS sorting

The use of human glioblastoma tissue samples and cell cultures were conducted in accordance with protocols approved by Melbourne Health Human Research and Ethics Committee (HREC number 2009.016). Cells were maintained in DMEM/F12 (Gibco) supplemented with 10ng/L EGF (bdBiosciences), 10 ng/L FGF (bdBiosciences), 1x B27 without vitamin A (Gibco) and 1x Penicillin/Streptomycin (Gibco). For flow cytometry sorting and analysis, cells were dissociated with accutase (BD Biosciences) and labeled with CD44-FITC (clone DB105, #130-098-210, Miltenyi-Biotec), CD133-APC (clone AC133, #130-098-829, Miltenyi-Biotec) and CD15-PE (SSEA-1, clone MC-480, #13-8813-82, eBioscience) along with the relevant isotype controls for gating (Figure S4A). Cells were sorted on a BD FACSAria III and analyzed on a BD LSRFortessa.

### Cell biology assays

For sphere forming assays, 50, 10, 5, 3 and 1 cells per well were plated into flat bottom, ultra low attachment 96 well plates (Corning). A minimum of 6 replicates per cellular dilution was used. Sphere forming efficiency was estimated by limiting dilution analysis using ELDA [62].

For cell invasion assays, 1000 cells were directly sorted onto round bottom, ultra low attachment 96 well plates (Corning) containing 100 μL media. 24 hours later an equal volume of Cultrex BME invasion matrix (Trevigen) was added and incubated for 1 hour at 37°C. An hour later 2 volumes of media was overlaid onto the invasion matrix. 7 days later the spheres were photographed and analyzed by ImageJ version 1.48. Briefly, a manual threshold was applied to mask cells, the image was segmented and the surface area occupied by cells quantified (Figure S4B). Surface area is represented relative to a media only sample to control for proliferation.

Temozolomide sensitivity was measured by sorting 1000 cells directly into flat bottom, ultra low attachment 96 well plates (Corning) containing 100 μL media. After 24 hours to allow sphere formation cells were dosed daily with 50 μM temozolomide (Sigma-Alrich) with a 50% media change [63]. Cell number after 7 days of drug exposure was measured by Resazurin assay (R & D systems). After addition of 10% v/v Resazurin, cells were incubated for 4 hours at 37°C. Cell number was estimated by fluorescence using an Enspire plate reader at 544 nm/590 nm excitation/emission.

### Statistical analyses

All analyses were conducted in R version 3.0.3 unless otherwise stated [59]. The survival package in R was used to construct a Kaplan Meier plot and build a Cox proportional hazards model [60]. The coin package was used to test for survival differences using a log ranked test [61].

## SUPPLEMENTARY MATERIAL, FIGURES AND TABLES






