# Supplementary material for: Coexpression analysis of CD133 and CD44 identifies Proneural and Mesenchymal subtypes of glioblastoma multiforme
Source: Oncotarget. 2015 Jan 31;6(8):6267–80. doi: 10.18632/oncotarget.3365 (PMC4467436; doi:10.18632/oncotarget.3365)
Supplement: Supplementary file 1 [file oncotarget-06-6267-s001.pdf]

Coexpression analysis of CD133 and CD44 identifies Proneural and Mesenchymal subtypes of glioblastoma multiforme

Supplementary Material

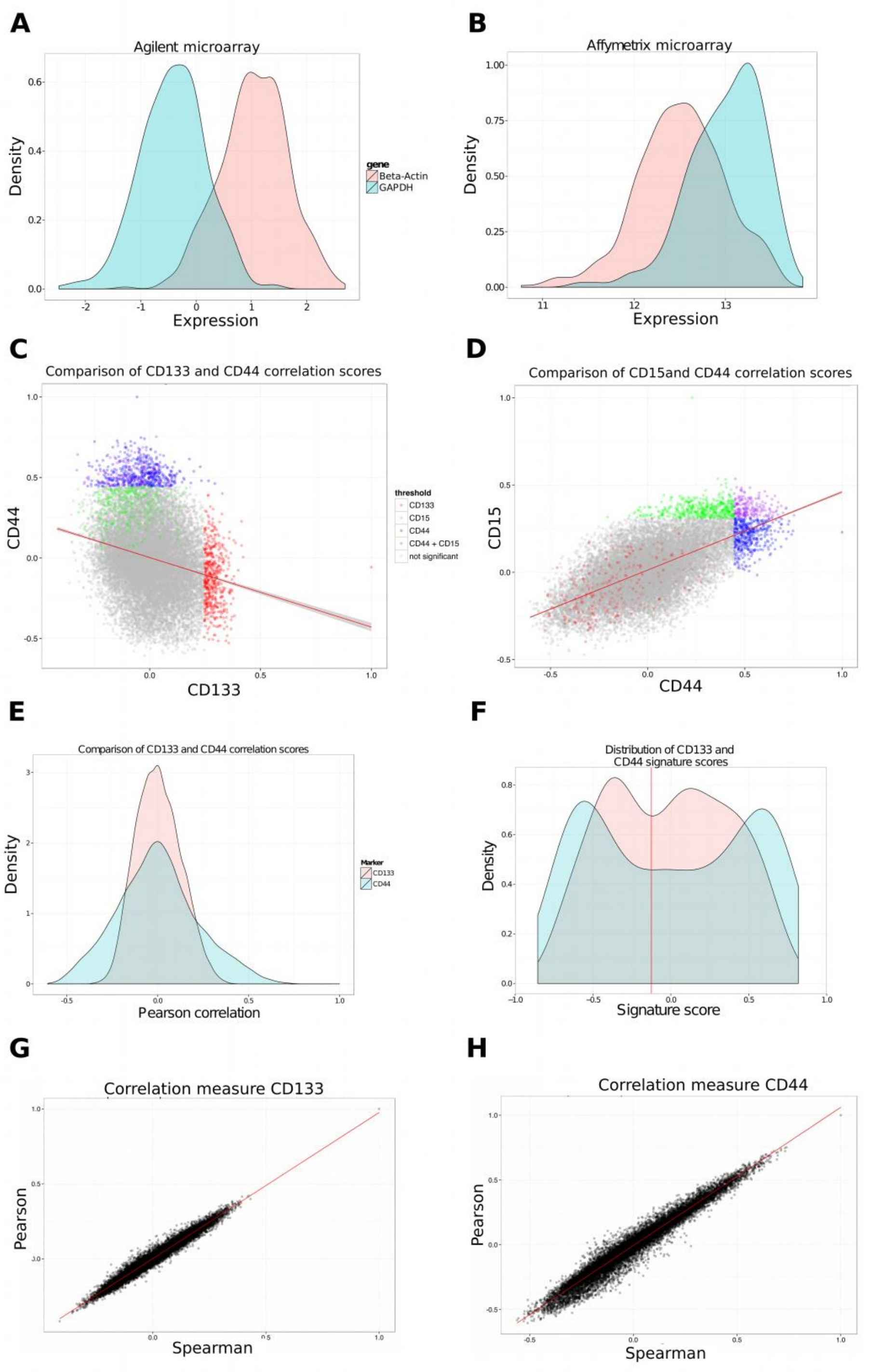

Figure S1: Exploration of the parameters used in the coexpression analysis.

Distribution of housekeeping gene expression. The intersect of the patients measured on both Agilent (A) and Affymetrix (B) platforms (n=443) was used to examine the expression of the beta-actin and GAPDH genes. (C) Negative relationship of the CD133 and CD44 Pearson correlation coefficients. Genes comprising marker coexpression modules are indicated by color. (D) Positive relationship of CD15 and CD44 Pearson correlation coefficients. (E) Histogram of the distribution of CD133 and CD44 correlated genes shows a greater magnitude for CD44. (F) Histogram of the distribution of single patient module scores for the CD133 and CD44 coexpression signatures. As for the correlation values, CD44 has a greater magnitude. Comparison of Pearson and Spearman correlation for CD133 (G) and CD44 (H).

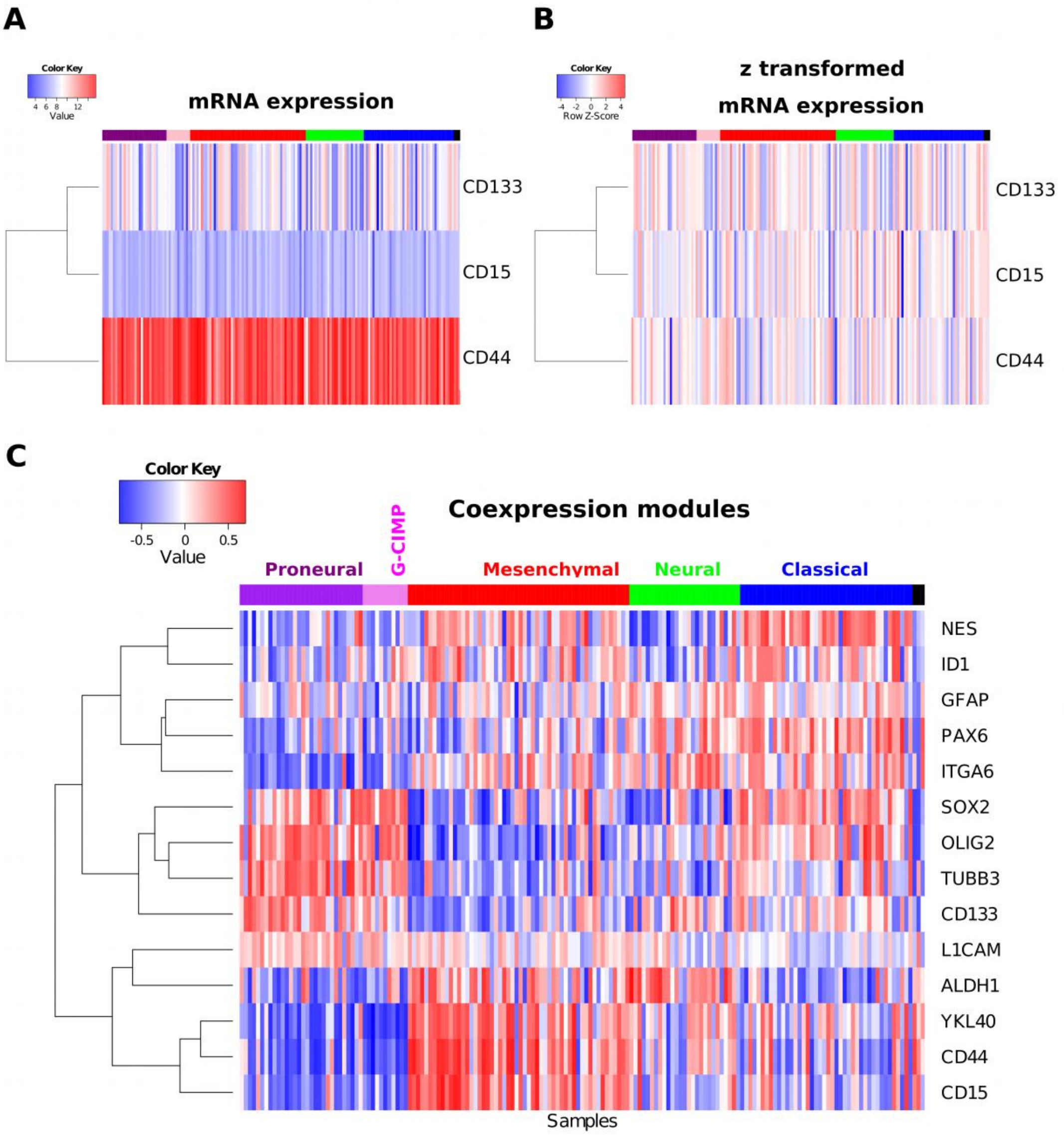

Figure S2: Additional markers for coexpression module analysis.

(A) Comparison of raw mRNA expression for CD133, CD15 and CD44. (B) z-score normalized mRNA expression for CD133, CD15 and CD44. (C) Additional intracellular markers of neural differentiation were added to the coexpression module analysis. 2 broad clusters corresponding to CD133 and CD44 signatures are apparent.

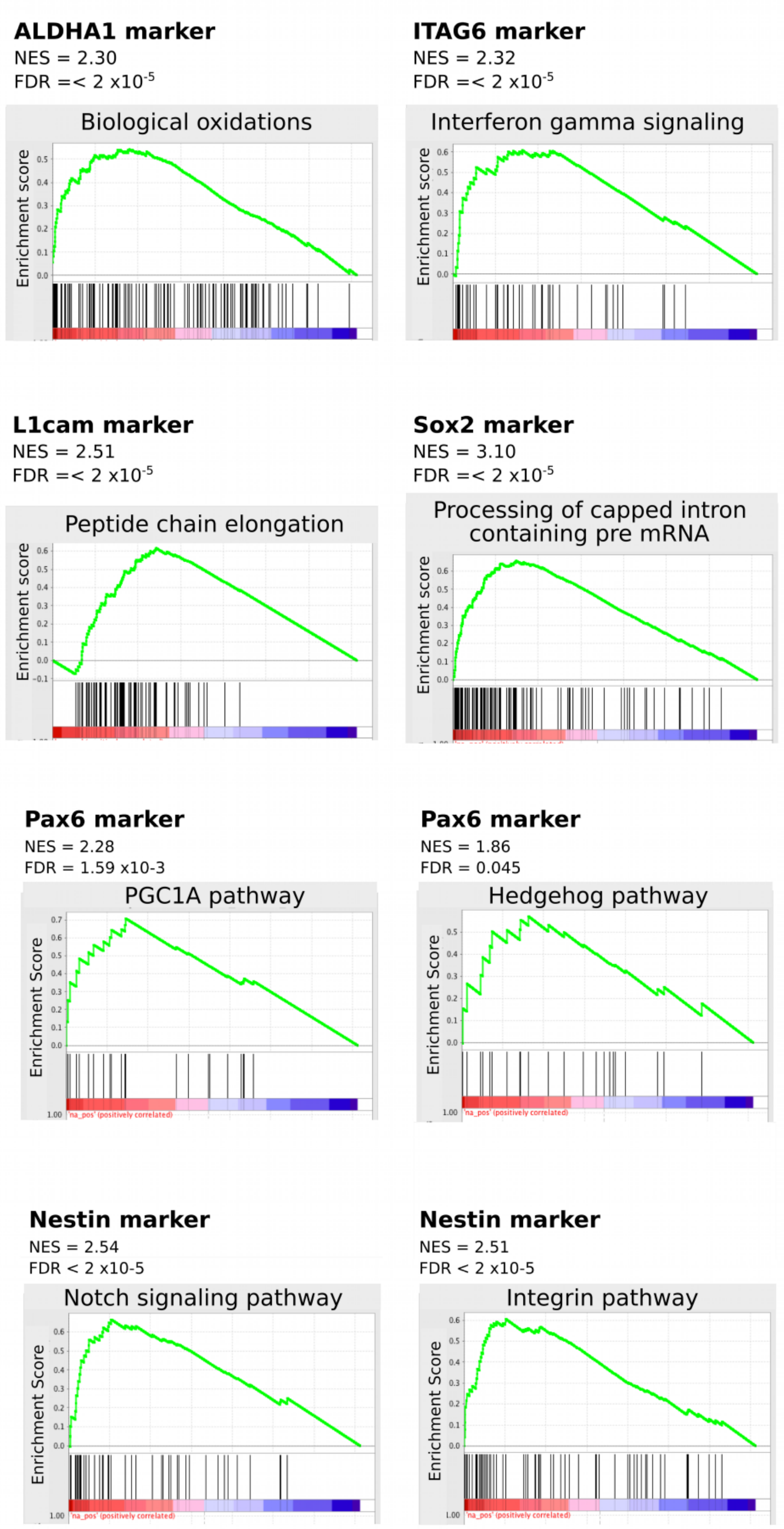

Figure S3: Pathways enriched in the coexpression modules of alternate cancer stem cell markers. The Pearson correlation coefficient of all genes with the marker of interest was used as input to GSEA in prereanked mode. The top pathway is shown for ALDHA1, ITAG6, L1CAM and Sox2. Two significant stem cell pathways are shown for Pax6 and Nestin.

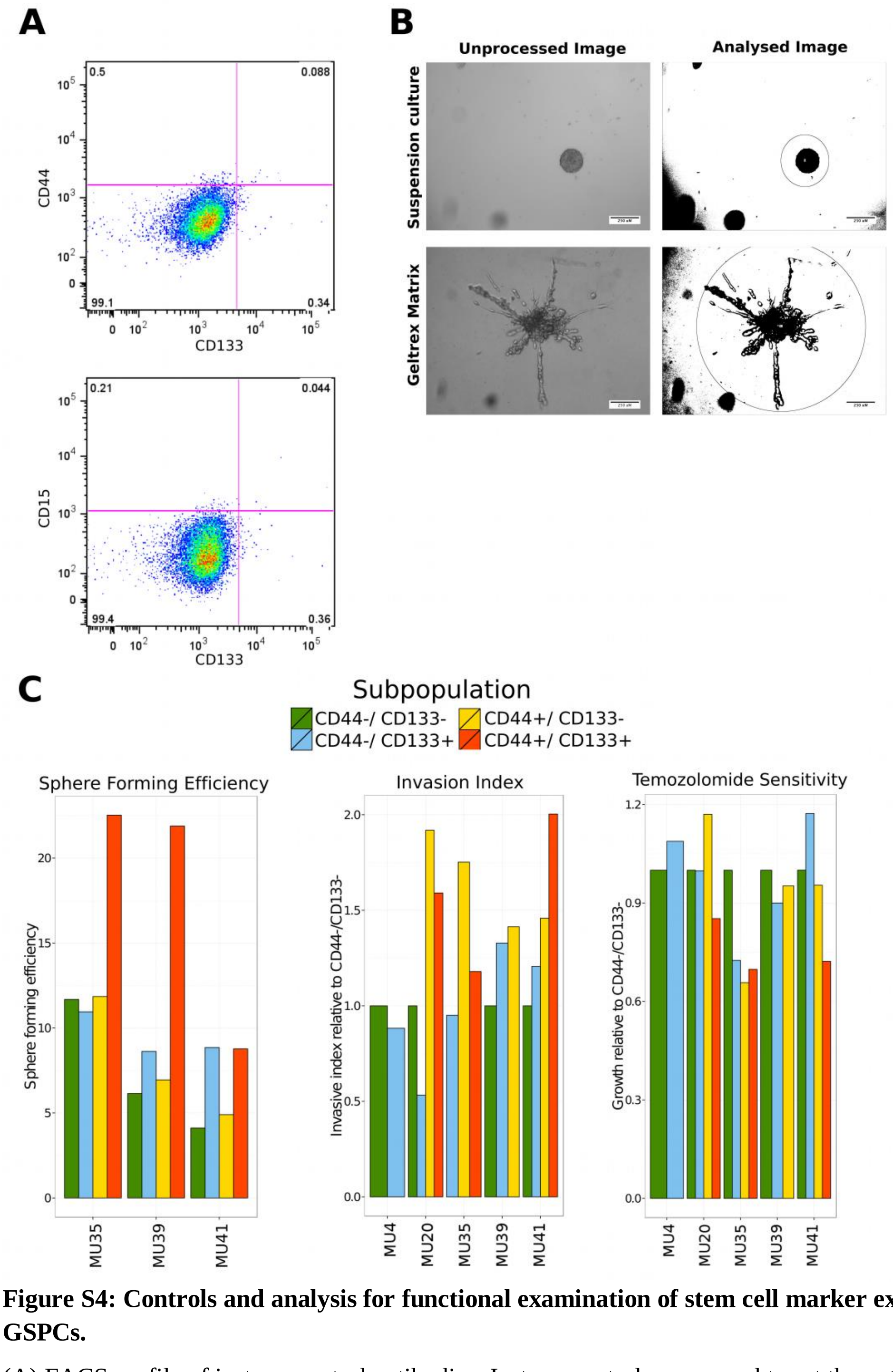

Figure S4: Controls and analysis for functional examination of stem cell marker expressing GSPCs.

(A) FACS profile of isotype control antibodies. Isotype controls were used to set the gate for the CD44-/ CD133- population. (B) Invasion assay raw and processed images. The surface area of the black masked cells within the enclosing circle was measured. The surface area of the suspension culture cells was subtracted from the matrix grown cells to control for proliferation. (C) Assay measurements for individual GSPC lines. Sphere forming efficiency (left), invasion (center) and temozolomide sensitivity (right).

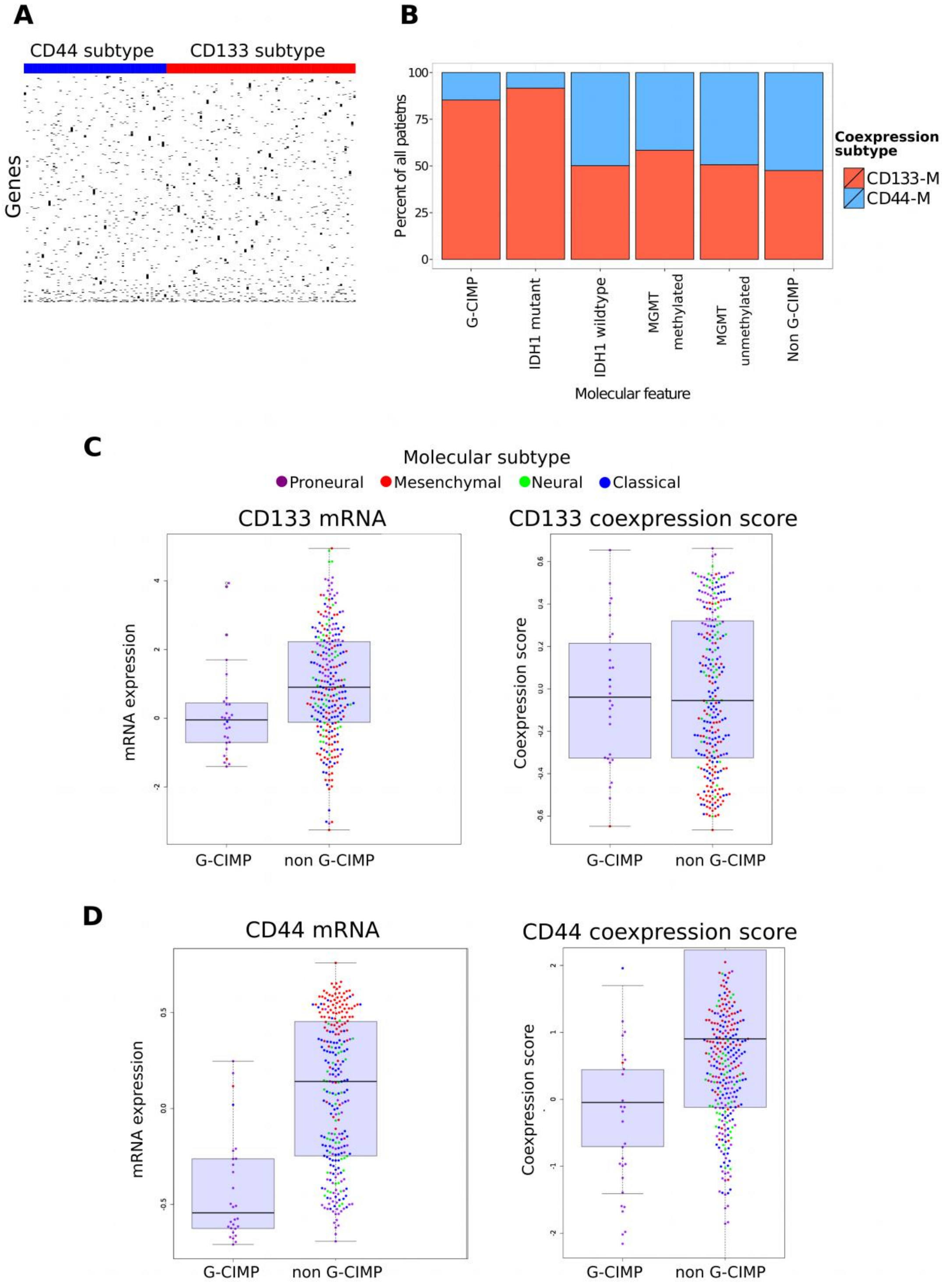

Figure S5: Integration of coexpression subtype with additional clinical features.

Patients were segmented into either CD133-M or CD44-M subtype and analyzed separately. (A) No difference in the total number of mutations. 200 of the most frequently mutated genes are represented. Black indicates mutated genes. (B) Comparison of clinical parameters across CD44-M and CD133-M subtypes. (C) Reduced expression of CD133 mRNA but not CD133-M subtype in G-CIMP samples. (D) Reduced expression of CD44 mRNA and CD44-M subtype in G-CIMP samples.

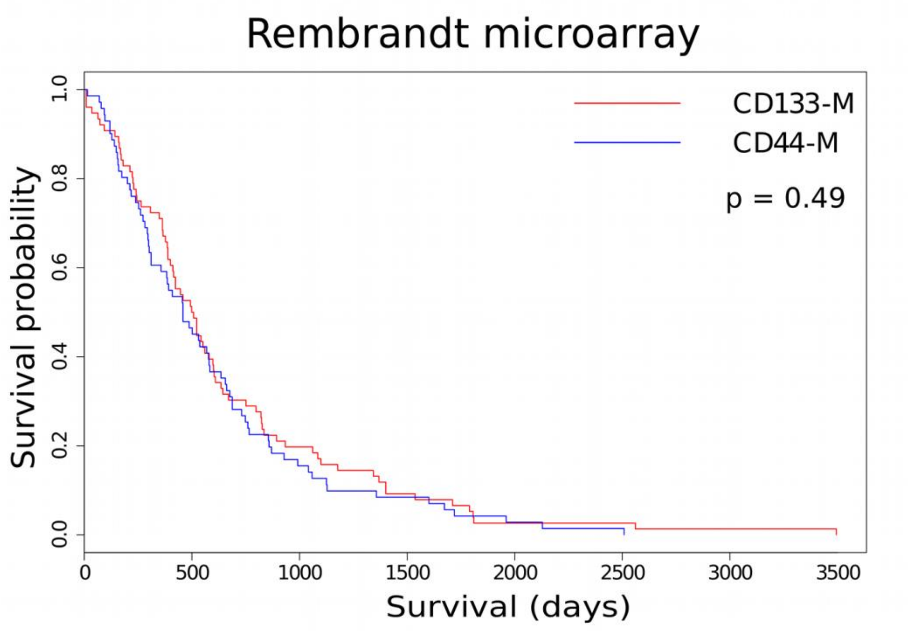

Figure S6: No significant difference in survival for CD44 and CD133 patients within the Rembrandt cohort.

Molecular subtype was assigned by GSVA using Verhaak et al. 2010 signatures.

Table S1: Genes comprising coexpression module signatures for stem cell markers.

Table S2: Genes common to both coexpression module signature and GBM molecular subtype signature from Verhaak et al., 2010.
